# Stenting in Non-Small Cell Lung Cancer: How Does It Affect the Outcomes?

**DOI:** 10.31557/APJCP.2020.21.1.175

**Published:** 2020

**Authors:** Nagla Abdel Karim, Sinan Khaddam, Mahmoud Shehata, Ahmed Mostafa, Mohamed Magdy, Ihab Eldessouki, Changchun Xie, Sadia Benzaquen

**Affiliations:** 1 *Hematology-Oncology Department Augusta University, *; 2 *Hematology-Oncology department, Vontz Center, University of Cincinnati, Ohio, U S A. *

**Keywords:** Stenting, non-small cell lung cancer, lung cancer, central airway obstruction

## Abstract

**Objective:**

Approximately 30% of lung cancer patients develop central airway obstruction (CAO) that remarkably shortens survival. There is little data about the benefits of stenting within this heterogeneous patient group. Our objective was to review their overall survival (OS) and their risk of hospitalization versus patients who did not have lesions requiring stent placement.

**Methods:**

We retrospectively reviewed charts of 171 non-small cell lung cancer (NSCLC) patients who underwent bronchoscopy in the University of Cincinnati Cancer Center from the year 2011 to 2013. Twenty-five patients with advanced lung cancer were evaluated by interventional pulmonology service for endobronchial stent placement for CAO. Eight patients did not require placement of a stent and 17 had obstructive lesions that required stenting by interventional pulmonology.

**Results:**

Demographical parameters such as age and gender did not have a significant impact on the risk of hospitalization or OS of both groups of patients, however, those whose lesions did not mandate stent placement had significantly lower odds of hospitalization compared to patients with CAO requiring a stent (OR: 15.913, 95% CI: 1.211-209.068, P = 0.0352). Patients with advanced NSCLC and CAO that required stent placement had an OS of 13.9 m [3.9-19.9 m] compared to an OS of 23.9 m for patients with CAO not requiring a stent. We found out that patients with less severe CAO have lower odds of hospitalization and better OS compared to patients with CAO mandating stent placement.

**Conclusion:**

CAO patients with interventional pulmonology (IP) evaluation and management in addition, may have improved OS suggesting that IP consultation might offer both improvement in quality of life and overall survival to patients with advanced NSCLC and CAO.

## Introduction

Central airway obstruction (CAO) mainly occurs secondary to malignant lesions and to a lesser extent due to benign lesions. Direct extension from an adjacent tumor, most commonly bronchogenic carcinoma, is the most common cause of malignant CAO (Ernst et al., 2002). Approximately 30% of lung cancer patients develop CAO, thus increasing the risk of post- obstructive pneumonia and respiratory failure. It is known that the development of CAO shortens survival remarkably; the literature reports 2-3 months survival if untreated, and 6-8 months with interventional treatment. 

Around 40% of lung cancer deaths can be attributed to locoregional disease (Noppen et al., 1997; Karim et al., 2017). Emergence of subspecialties in interventional techniques such as interventional pulmonology resulted in a surge in research directed toward treating patients with complex airway pathology. The main objectives was to improve the quality of life (QoL) for these patients and to decrease the need for hospitalizations and surgeries especially for patients who are not eligible for the latter (Ernst et al., 2002). Therapeutic interventional bronchoscopy such as airway stenting (AS) in particular was found that it may provide immediate and effective palliation resulting in improvement in the patient’s QoL. The idea of airway stenting appeared with Trendelenburg and Bond in the late 1800s. The first reference to a polyethylene tube to act as a stent was in 1954 but the use of the word “stent” was not popular until the 1980s, when stents became widely used in different vascular, urologic, and biliary procedures (Sterioff, 1997). Jean-François Dumon introduced the first completely endoluminal airway stent in 19908. Three main types of malignant airway obstruction were described: endobronchial obstruction, extrinsic compression, and mixed pattern. Ablative techniques that destroy tissue are indicated for endobronchial obstruction while stents are used in extrinsic compression so as to strengthen the bronchial wall and keep the airway open. And for mixed patterns, ablation followed by stenting is usually adopted (Ost et al., 2015). 

While many investigators think that it would be unethical to randomize patients with CAO with symptomatic disease to therapeutic procedures, it may be impossible to adopt double-blinding in this population. For these reasons, literature is mainly made up of case series and retrospective analyses. Nevertheless, the impact of therapy on both QoL and survival is remarkable, and with the availability of the new techniques and procedures, the prospective of treatment will change. There will be no doubt whether treatment is helpful or not, the only remaining question will be which technique should be used for which patient in particular (Ernst et al., 2002). Answering that question will require many studies comparing different modalities and different subgroups within the patients with the target pathology. Our study represents one of these studies. 

A consensus has been made that a symptomatic CAO who present with an extraluminal airway stenosis with ≥50% airway narrowing or partially intrinsic airway stenosis with ≥50% airway narrowing after debulking require stenting (Verma et al., 2017). In 2007, a standardized classification scheme was proposed for rapid and uniform classification of central airway stenosis (Verma et al., 2017). There is not much data about the impact on OS or the risk of hospitalization in CAO patients mandating stent placement compared to those who did not require stenting. Most previous studies compared outcome in patients with CAO and patient without. While patients with CAO not requiring stent placement were excluded in previous studies, to our knowledge, this is the first study to explore the outcome of patients with two types of obstructive airway lesions based on whether they mandate a stent or not. 

## Materials and Methods

*Patient Population*

The charts of 171 patients who underwent bronchoscopy from the year of 2011 to 2013 were reviewed. We also reviewed the pathological diagnosis, LN stations, PET scan results and the endobronchial ultrasound (EBUS) operators. 

*Eligibility*

Twenty-five patients with advanced lung cancer were evaluated by the interventional pulmonary [IP] service at the University of Cincinnati for endobronchial stent placement for CAO between 2011- 2013. Stent placement was indicated if the patient had more than 50% extrinsic compression of a main airway (trachea, RMS or LMS bronchus), the patient had to be symptomatic while the distal airway should be patent. Seventeen patients had obstructive lesions that required stenting while 8 patients did not require placement of a stent.

*Statistical Analysis*

Death was considered as the endpoint. Kaplan-Meier method was used to calculate median overall survival and 95% CI. Cox model was used to test the overall survival difference between the patients who need stent and patients who do not need stent adjusted for age and sex. Logistic regression was used to test the hospitalization rate difference between the patients who need stent and patients who do not need stent adjusted for age and sex. Data were analyzed using the SAS^®^ Version 9.4.

## Results

The eight patients whose lesions did not need stent placement had significantly lower chances of hospitalization compared to the other 17 patients with CAO requiring a stent (OR:15.9, 95%CI:1.211~209.068, P = 0.0352). Patients with advanced NSCLC and CAO who needed IP stent placement had an OS of 13.9m (range, 3.9-19.9m) compared to 23.9m OS (range, 426- upper limit could not identified due to small sample number) for patients with CAO did not need a stent. 

## Discussion

We compared the results of our retrospective study to previous data available from 1990 till 2017 in PubMed and google scholar. Our study suggests that survival and length of hospitalization highly depend on the degree of obstruction. Patients with CAO who did not require stent placement had a lower risk of hospitalization and better overall survival when compared to patients with CAO who needed stent placement. Despite the fact that stenting in patients with the more significant stenosis did not improve survival to be similar to those not requiring stenting, they still had better survival than expected if stenting was not offered. Our results are consistent with previous studies stating that stenting is averting premature death, thus allowing application of cancer targeted therapy and restoring impending shortened survival to expected life expectancy associated with the underlying malignancy (Ernst et al., 2002; Chhajed et al., 2006). This approach was particularly significant in lung cancer patients with tumors of aggressive nature and late presentations (Karim et al., 2018; Eldessouki et al., 2018; Karim et al., 2017; Eldessouki et al., 2018). The overall survival of 13.9m in our study is slightly better than expected in patients with advanced NSCLC. That suggests improved outcome in patients with advanced NSCLC who were offered the current interventional pulmonology procedures integrated in the current multimodality treatment. Also, the survival in patients with CAO in this study is better than what was reported in many older studies investigating the same disease, which may be related to the recent advancement in adjuvant therapy including immunotherapy, which became possible to more patients with CAO by preventing their premature death with the help of IP procedures like stenting (Chhajed et al., 2006; Hassan et al., 2017; Karim et al., 2017; Karim et al., 2018). Our results are in the context of old studies which suggested that combined therapeutic bronchoscopy and chemotherapy should be actively considerable in all patients with lung cancer and central airway obstruction (Noppen et al., 1997). Evaluation and management of patients with CAO require a thorough knowledge of the etiology, physiology, diagnostic, and treatment options, as well as a multidisciplinary team approach including chest radiologists, anesthesiologists, medical oncologists, thoracic surgeons, and interventional pulmonologists (Ernst et al., 2004). As per the results of the AQuIRE registry study, the results of stents varied significantly between centers. Among the 8 centers with data on 25 cases, the proportion of cases in which a stent was placed ranged from 13% to 69% (P,.001) (Ost et al., 2015). That study showed that 30-day mortality was higher in patients who had stents placed. However, the association between stents and 30-day mortality may be the result of residual confounding or confounding by indication. Specifically, patients who required stents may have had a higher disease burden, and therefore, the observed decrease in survival might be due to these unaccounted-for factors. This aforementioned interpretation is more consistent with our results. Since patients with more severe CAO disease are those having stents placement, then they also have worse survival possibly due to the more locally advanced disease nature rather than due to having a stent placed, specially that in our study some improvement in survival was noticed in those groups of patients with more severe CAO after stent placement. Contributing researchers to the study who used the results of AQuIRE registry believed that selectively stenting only patients who really require it to be prudent. That agrees with the suggestions of our study, though our study still did not compare stenting vs no stenting in asymptomatic patients. That last comparison was reported before in SPOC trial. It investigated the role of a silicone prosthesis in preventing airway obstruction recurrence in asymptomatic residual CAO <50% of the normal; and it could confirm improved (HR 0.71; P=0.17) recurrence free survival and benefit on dyspnea (P=0.08) (Ernst et al., 2004; ERS, 2013). The role of stents in asymptomatic central airway lesions may be the focus of future clinical trial, as that does not carry ethical challenges that prevents randomizing patients with severe symptoms. 

The costs of these interventional pulmonology therapies must also be balanced by their potential cost savings in terms of overall health care dollars. Numerous studies have shown that therapeutic bronchoscopy resulted in an instant reduction in the level of care, and was associated with the ability to release patients from mechanical ventilation. The additional costs, therefore, may be offset by a decrease in intensive care unit, and hospital length of stay (ERS, 2013). An example is a study by Colt and Harrell that reviewed the records of 32 patients with CAO requiring admission to an intensive care unit before therapeutic intervention with a rigid scope. Following therapeutic rigid endoscopy, 20 of these patients had an immediate reduction in the level of care. On the other hand, in the results of AQuIRE registry study, the data suggested that over the first 24 hours, therapeutic bronchoscopy occasionally results in an increase in level of care required, although possibly over the long term, quality of life and level of care needed improve (Ost et al., 2015). Our study which focused specifically on placement of stents suggested that stents as an interventional pulmonology therapeutic procedure improved symptom, quality of life, and also downgraded the level of care inpatient. 

Previous studies that also focused on symptoms and functional status results of stents placement, reported improvement in the mean preoperative MRC dyspnea scale score and the mean preoperative ECOG performance status (Razi et al., 2010). In regionally advanced cancer patients, the palliative nature of this procedure postpones death by respiratory distress and may also prompt consideration for institution of conservative comfort measures to reduce patient suffering (ERS, 2013).

In conclusion, lung cancer patients with CAO who do not require a stent per the current criteria, have a lower risk of hospitalization and have better OS compared to patients with CAO mandating stent placement. However, CAO patients with interventional pulmonology evaluation and management in addition, may have improved OS suggesting that IP consultation may offer improvement in quality of life and overall survival to patients with advanced NSCLC and CAO. 
